# Inhibition of Neuron-Restrictive Silencing Factor (REST/NRSF) Chromatin Binding Attenuates Epileptogenesis

**DOI:** 10.1523/ENEURO.0006-24.2024

**Published:** 2024-05-17

**Authors:** Alicia M. Hall, Noriko Kamei, Manlin Shao, Hyun-Seung Mun, Kevin Chen, Yuncai Chen, Tallie Z. Baram

**Affiliations:** ^1^Department of Pediatrics, University of California-Irvine, Irvine, California 92697; ^2^Department of Anatomy and Neurobiology, University of California-Irvine, Irvine, California 92697; ^3^Department of Neurology, University of California-Irvine, Irvine, California 92697

**Keywords:** epigenetic, epileptogenesis, kainic acid, REST/NRSF, status epilepticus, transcription factor

## Abstract

The mechanisms by which brain insults lead to subsequent epilepsy remain unclear. Insults including trauma, stroke, infections, and long seizures (status epilepticus, SE) increase the nuclear expression and chromatin binding of the neuron-restrictive silencing factor/RE-1 silencing transcription factor (NRSF/REST). REST/NRSF orchestrates major disruption of the expression of key neuronal genes, including ion channels and neurotransmitter receptors, potentially contributing to epileptogenesis. Accordingly, transient interference with REST/NRSF chromatin binding after an epilepsy-provoking SE suppressed spontaneous seizures for the 12 d duration of a prior study. However, whether the onset of epileptogenesis was suppressed or only delayed has remained unresolved. The current experiments determined if transient interference with REST/NRSF chromatin binding prevented epileptogenesis enduringly or, alternatively, slowed epilepsy onset. Epileptogenesis was elicited in adult male rats via systemic kainic acid-induced SE (KA-SE). We then determined if decoy, NRSF-binding–motif oligodeoxynucleotides (NRSE-ODNs), given twice following KA-SE (1) prevented REST/NRSF binding to chromatin, using chromatin immunoprecipitation, or (2) prevented the onset of spontaneous seizures, measured with chronic digital video-electroencephalogram. Blocking NRSF function transiently after KA-SE significantly lengthened the latent period to a first spontaneous seizure. Whereas this intervention did not influence the duration and severity of spontaneous seizures, total seizure number and seizure burden were lower in the NRSE-ODN compared with scrambled-ODN cohorts. Transient interference with REST/NRSF function after KA-SE delays and moderately attenuates insult-related hippocampal epilepsy, but does not abolish it. Thus, the anticonvulsant and antiepileptogenic actions of NRSF are but one of the multifactorial mechanisms generating epilepsy in the adult brain.

## Significance Statement

The mechanisms by which brain insults can lead to subsequent epilepsy remain unclear. Insults may influence neuronal functions by enduringly changing their gene expression programs, often via changes in master regulators such as transcription factors (TFs). The TF REST/NRSF is activated by insults, alters gene expression selectively, and thus promotes aberrant neuronal function and connectivity. Previously, blocking REST/NRSF function transiently in a developing brain prevented cognitive problems that accompany status epilepticus (SE)-induced epilepsy. Here, blocking REST/NRSF DNA binding transiently following SE in adult rats delayed and attenuated epileptogenesis, but did not abolish it.

## Introduction

Epilepsy, the generation of apparently spontaneous seizures, is the third most common chronic brain disorder and exerts an enormous toll on human potential (Committee, 2012). The epilepsies may derive from genetic factors, including increasingly recognized gene mutations (EpiPM, 2015; Helbig et al., 2016, 2018), or arise in the context of prior insults such as traumatic brain injury (Pitkänen and Immonen, 2014), brain infection (Patel et al., 2017), or stroke (Chang et al., 2022; Phan et al., 2022). Because of the clear and causal association of insults and epileptogenesis, uncovering the mechanisms leading to an insult to the onset of epilepsy and the commonly associated cognitive and emotional problems is critical for the identification of preventative and therapeutic targets (Committee, 2012).

Experimental models of insult-related epilepsy, including those involving the generation of inciting prolonged seizures (status epilepticus, SE), have contributed greatly to the discovery of epileptogenic mechanisms (Löscher, 2012). These mechanisms include triggering of innate immune/inflammatory processes which disrupt neuronal and circuit activity (Vezzani et al., 2011), as well as enduring changes in neuronal and circuit functions triggered by persistent changes in the repertoire of genes they express (Roopra et al., 2012; Hauser et al., 2018; Kobow et al., 2020). Several key transcriptional factors and pathways have been directly implicated in epileptogenesis, including TGFβ (Friedman et al., 2014), JAK/STAT (Raible et al., 2015), and the neuron-restrictive silencing factor REST/NRSF (Garriga-Canut et al., 2006; Bassuk et al., 2008; McClelland et al., 2011a, 2014; Brennan et al., 2016; Hwang and Zukin, 2018; Navarrette-Modesto et al., 2019; Prestigio et al., 2021).

In adult rats that developed epilepsy following SE provoked by the convulsant kainic acid (KA), the repression of ∼3 doz of key neuronal genes was identified, including the ion channel HCN1 and glutamate and GABA receptor subunits (McClelland et al., 2011a, 2014).

REST/NRSF was identified as a key orchestrator of these gene changes: REST/NRSF nuclear expression was augmented after SE for about a week (McClelland et al., 2014), and transiently blocking REST/NRSF function (i.e., its binding to the chromatin) prevented the onset of spontaneous seizures during the first 10 d following SE (McClelland et al., 2011a). In addition, in immature rats experiencing SE provoked by experimental fever, a subsequent transient block of REST/NRSF prevented SE-induced cognitive problems in adulthood (Patterson et al., 2017). Together, these studies suggested that NRSF is a key contributor to transcriptional changes induced by SE, and these changes may underlie epileptogenesis and/or cognitive comorbidities of epilepsy. Furthermore, transient interference with the binding of REST/NRSF to the chromatin suffices to prevent memory problems associated with epileptogenesis in immature animals. However, because of the short duration of recording in adult rats following KA-induced SE (KA-SE), it remained unclear whether blocking REST/NRSF suppressed the onset of spontaneous seizures transiently or, instead, attenuated epileptogenesis long term. The current studies address this question.

## Materials and Methods

### Animals and experimental design

**Figure 1.**
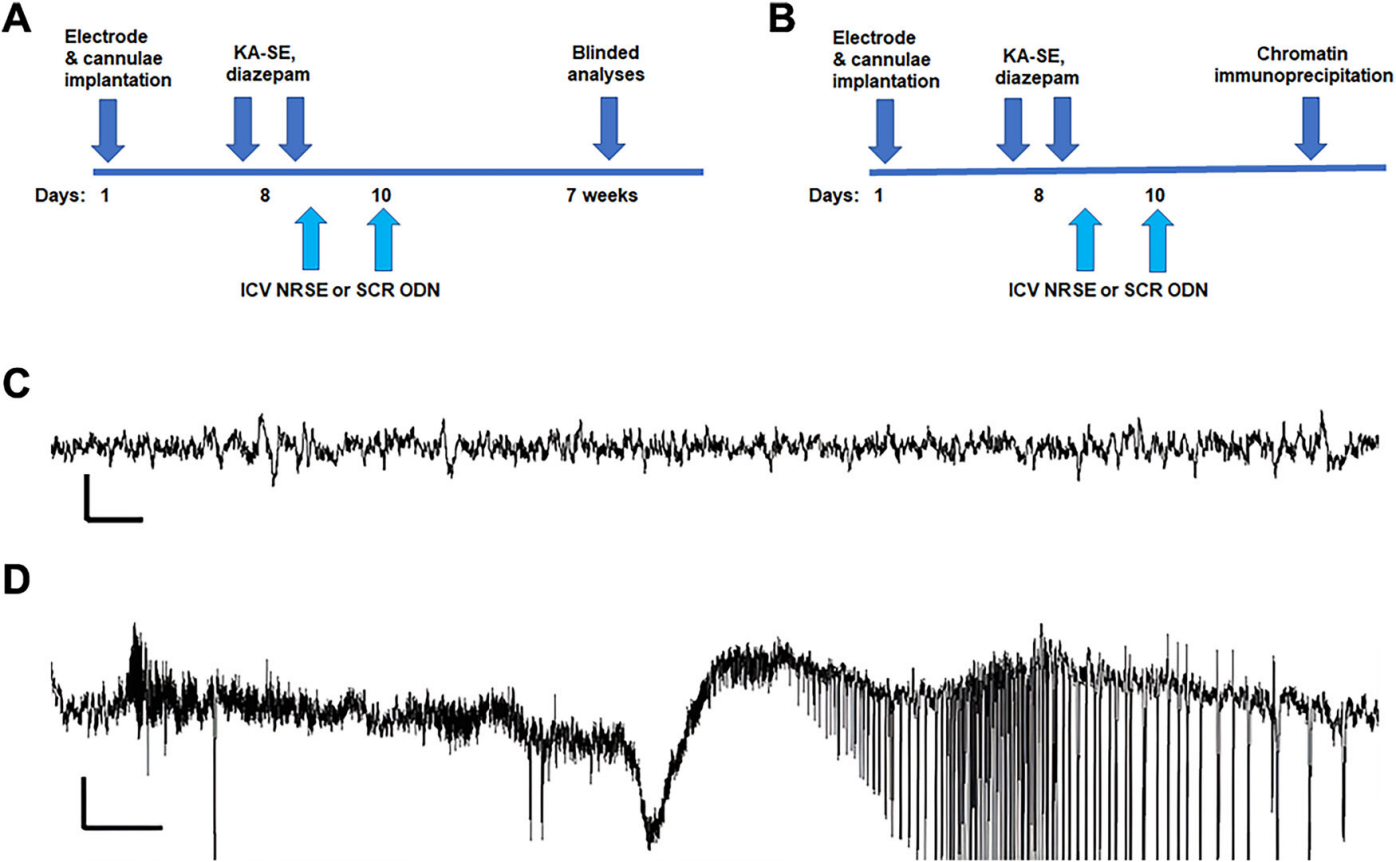
Experimental design and sample EEGs. Two rat cohorts were employed. ***A***, The first cohort experienced KA-SE (*n* = 21) or served as controls. KA-SE rats were then randomized to receive either a SCR-ODN or an ODN corresponding to the REST/NRSF binding motif of DNA (NRSE-ODN) immediately following the SE as well as 2 d later. Rats were recorded chronically, and video-EEGs from these rats were analyzed without the knowledge of the group. ***B***, A second cohort underwent the same KA-SE procedure or served as controls, then received the ODNs, and were killed 48 or 72 h later for ChIP. ***C***, Sample EEG recorded from bipolar hippocampal electrodes showing typical hippocampal baseline rhythms. Note expanded scale: horizontal bar, 1 s; vertical bar, 200 microvolts. ***D***, Hippocampal EEG from a KA-SE rat that received SCR-ODN. A typical baseline is interrupted by spikes and then evolves into a seizure. The same pattern was observed in KA-SE rats receiving NRSE-ODNs. Scale, horizontal, 1 s; vertical 500 microvolts.

Adult (∼2 month old) male Sprague Dawley rats (Harlan; RRID:RGD_5508397) were used. They were housed under a 12 h light–dark cycle in quiet rooms with *ad libitum* access to food and water. The experimental design is schematized in Figure 1. We included four groups: controls receiving scrambled (SCR) oligodeoxynucleotide (ODN), controls receiving NRSE-ODN, KA-SE receiving SCR-ODNs, and KA-SE receiving NRSE-ODNs. The primary outcome measures were the presence or absence of spontaneous seizures, the latency to their onset, and their frequency and durations. We calculated group sizes using power analysis (https://select-statistics.co.uk/calculators/sample-size-calculator-two-means/) and aimed for *n* = 11/group for the KA-SE groups and *n* = 5 for controls.

To assess the chromatin binding of NRSF/REST, a separate group of male rats (*n* = 4 per group, based on effect sizes in McClelland et al. (2011a, 2014) served as controls or underwent KA-SE with the administration of either SCR- or NRSE-ODNs. These rats were killed 48 h (control and KA-SE) or 72 h (all groups) after the end of the SE (receiving two doses of the ODN); times were shown to be associated with high levels of SE-induced REST/NRSF hippocampal levels and binding to the chromatin. The 48 and 72 h groups were combined, and we lost one of the KA-SE-NRSE-ODN rats resulting in sample sizes of *n* = 3–8.

None of the controls developed any measurable seizures; thus, the results reported here are primarily from the KA-SE-SCR and KA-SE-NRSE cohorts, except for control rats used for the chromatin immunoprecipitation (ChIP). Two SCR rats and one NRSE rat were excluded for technical reasons including the loss of electroencephalogram (EEG) head cap. All experiments were approved by the University Institutional Animal Care and Use Committee and conformed to NIH guidelines. We made all efforts to minimize the number and suffering of animals.

### Surgery

We anaesthetized adult rats weighing ∼250 g, two control groups (*n* = 5/group) and two KA-SE groups (*n* = 11–12/group) with 4% isoflurane. Skulls were shaved and placed into a stereotaxic frame, isoflurane levels were maintained using 2–3%, and eyes were hydrated using GenTeal. We treated the skin with iodine and ethanol and then made a midline scalp incision to expose the skull. We positioned the bilateral infusion cannula on the cortical surface (−1.0 mm posterior, ±1.5 mm lateral from the bregma) directly above the lateral ventricles, using the coordinates of Paxinos and Watson (Paxinos and Watson, 1982). We implanted bipolar electrodes (Plastics One) bilaterally into the hippocampus (AP, 3.3 mm; ML, 2.3 mm; DV, −2.8 mm from the bregma). For the tethered system, we secured two reference skull screws above the frontal cortex and two more skull screws to anchor the head cap. We encased the electrodes, cannula, and screws in the dental cement. For the remote system, we created a subcutaneous pocket using large scissors and a blunt dissection technique to make a pocket from the scalp to the left shoulder. Then, we inserted the implantable small animal CNS telemetry probe (Data Science International (DSI); model F40-EET) within the pocket and sutured the incision. Rats received 5 ml of 0.9% saline via subcutaneous injection to rehydrate and aid in recovery from surgery. We monitored the rats and allowed them 5–7 d of postsurgical recovery.

### Induction of SE

We induced SE using a published protocol (McClelland et al., 2011a; Brennan et al., 2016), following the procedure devised by Heller and Dudek (Hellier and Dudek, 2006). Briefly, intraperitoneal injections of 5 mg/kg of KA (Abcam; catalog #ab120100) were repeated to reach and maintain SE for 3 h (two rats received half doses, 2.5 mg/kg). We continuously monitored and scored seizures using the Racine scale (Racine, 1972). After 3 h of SE, KA-SE rats received a 8 mg/kg dose of diazepam (Patterson Veterinary) to terminate SE. We monitored the rats' behavior for overt seizure activity and gave another diazepam dose if necessary. We gave rats a subcutaneous injection of 5 ml of 0.9% saline for rehydration and provided a soft food diet for 3 d.

### Intracerebroventricular infusions

To eliminate the potential anticonvulsant/antiepileptic effects of the NRSE intervention, we administered the intervention after the proepileptogenic insult was over. Specifically, as described above, we generated a 3 h SE in all animals and then terminated the SE in all rats using diazepam, ascertaining an equal insult in all groups. Only then did we infuse the NRSE intervention as described below.

Thus, immediately following the termination of the SE using diazepam, we anesthetized the rats with 4% isoflurane and maintained anesthesia with 2.5%. We then infused either a SCR-ODN (AGGTCGTACGTTAATCGTCGC) or an active probe consisting of the DNA sequence of the DNA REST/NRSF binding site (NRSE) into the lateral ventricle of each hemisphere of the brain (McClelland et al., 2011a). The structure of the NRSE-ODN was designed to mimic the DNA binding site for REST/NRSF and function as a decoy, that is bind cellular REST/NRSF and prevent its chromatin binding: GGAGCTGTCCACAGTTCTGAA. Both ODNs were protected from degradation by substituting phosphorothioates for the phosphate backbone (Sigma-Aldrich). Specifically, the cannula needle was lowered so that the tip was within the lateral ventricle, and we infused 5 nmol/ventricle (5 µl at 1 nmol/µl) of NRSE-ODNs or SCR-ODNs at a rate of 0.5 µl/min once on the day of KA-SE and once 2 d later.

### Digital video-EEG recording and analyses

Following SE, we began video-EEG monitoring: EEG recording was synchronized to the video and conducted continuously for up to 2 months. We used a tethered system [ADInstruments; PowerLab data acquisition hardware and bioamplifiers and Labchart 7 software; Labchart 7 EEG recording required manual synchronization with Logitech Dynex (DX-NW080) video webcam] or an implantable telemetry system (DSI) for EEG acquisition and employed the same software for seizure detection and analysis (NeuroScore version 3.0). In post hoc analyses, there were no differences between the tethered and implantable systems in any parameter. In addition, two experienced investigators blind to the group identity visually scanned the coded EEGs for seizures (Dubé et al., 2010). Any suspicious activity led to the analysis of concurrent EEG and video recordings to identify behavioral manifestations of the apparent seizure. Only events with both EEG and behavioral changes that lasted over 10 s were classified as seizures. We evaluated typical behaviors associated with limbic-onset seizures, including sudden cessation of activity, facial automatisms, head bobbing, and prolonged immobility with staring. These progressed to alternating or bilateral clonus, rearing, and falling (Racine, 1972). Rats were considered epileptic if they had at least one documented seizure as defined above.

### ChIP

We anesthetized and decapitated the KA-SE rats 48 or 72 h after SE termination and isolated hippocampi. The right hippocampus was cross-linked with 37% formalin for 10 min at room temperature in PBS, and the cross-linking was neutralized by adding 1.4 M glycine. The pelleted tissue was dissociated in homogenization buffer (50 mM HEPES, pH 8.0, 140 mM NaCl, 1 mM EDTA, 0.4% IGEPAL CA-630, 0.2% Triton X-100, and a cocktail of protease inhibitors) and in a nuclear wash buffer (20 M mM Tris–Cl, pH 8.0, 0.15 M NaCl, and 1 mM EDTA). Nuclei collection was aided by centrifugation, and the nuclei were then sonicated for 10 min using a Diagenode Bioruptor. After removing cellular debris, the supernatant was precleared overnight with Protein A/G beads (Santa Cruz Biotechnology; catalog #sc-2003) at 4°C and incubated with 10 µg of either control nonimmune serum (IgG; Cell Signaling Technology; catalog #2729S, RRID:AB_1031062) or anti-REST/NRSF (Santa Cruz Biotechnology) overnight at 4°C in a buffer containing 16.7 mM Tris–Cl, pH 8.0, 167 mM NaCl, 1.2 mM EDTA, 1.1% Triton X-100, and protease inhibitors. Protein A/G beads blocked with salmon sperm DNA (400 µg/ml) and BSA (400 µg/ml) were added to the lysate for 2 h. Beads were washed twice with a low-salt wash buffer, high-salt wash buffer, LiCl wash buffer, and TE to remove nonspecifically bound protein and then eluted using a buffer containing 2% SDS and 0.2 M sodium carbonate. Eluates were reverse cross-linked at 65°C overnight, and the bound DNA was purified using the QIAquick MinElute PCR purification kit (Qiagen; catalog #28104). We performed quantitative PCR amplification using SYBR Green chemistry (Roche) on a LightCycler 96 (Roche) with primers specific for the NRSE/REST region of the HCN1 target gene. *HCN1* primer sequences were as follows (5′-3′): forward, AGGGAGCTGTCCACAGTTCTGAAT, and reverse, AAGTCCTTCAGTGGGGTTTTC. We report antibody binding to the gene as the percent input, calculated from average triplicate cycle threshold input values for each sample and the REST/NRSF ChIP after subtracting nonspecific binding assessed with IgG.

### Analyses and statistical considerations

All analyses were conducted without the knowledge of the experimental group. Group sizes were determined a priori during the experimental design phase based on power analyses, and animals were assigned to groups randomly. Statistical analyses were performed using the GraphPad Prism (RRID:SCR_002798) software, and all data are expressed as mean ± standard error of the mean (SEM), unless otherwise stated (Table 1). Unpaired Student's *t* test was used to evaluate the difference between two groups, and ANOVA was used for the three groups. Error bars indicate SEM. Outliers were excluded using the GraphPad Prism ROUT test for outliers.

**Table 1.** Statistical analysis

Data structure	Type of test	Power
Experiment 1: normal distribution	Latency: *t* test	See estimation plot, Figure 3*D*
Experiment 2: small group size	One-way ANOVA	*F*(2,9) = 6.033; *p* = 0.02

## Results

### SE augments REST/NRSF chromatin binding, and administration of “decoy” ODNs consisting of the REST/NRSF binding motif attenuates REST/NRSF this binding

**Figure 2.**
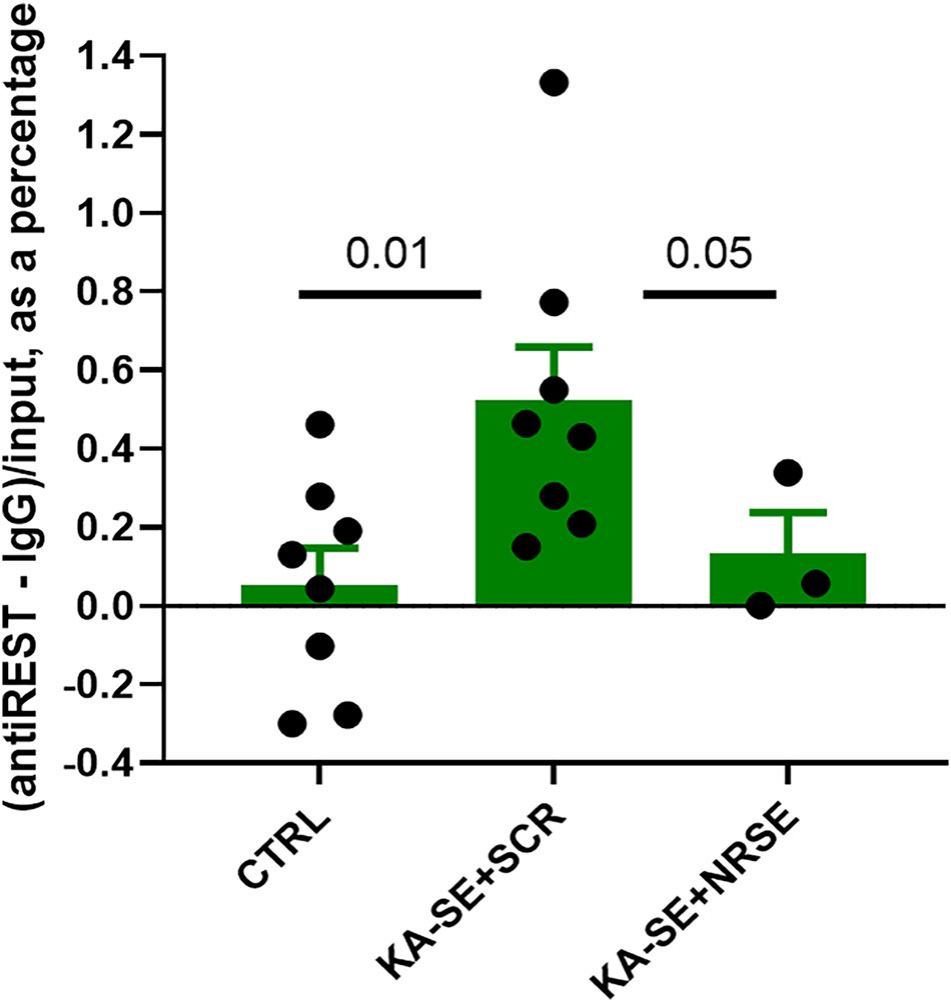
ChIP of hippocampi of control and two groups of KA-SE rats. The right hippocampus of control male rats (*n* = 8) and those experiencing KA-SA (*n* = 11) were obtained 48 or 72 h after termination of the insult. ChIP for the binding of REST/NRSF to the chromatin was performed as described in the Materials and Methods, ChIP. REST/NRSF chromatin binding was significantly different among groups (*F *= 5.19; *p *= 0.042; one-way ANOVA). The REST/NRSF chromatin binding was augmented in KA-SE rats receiving SCR-ODN, compared with controls (*p *= 0.017), and to rats receiving ODN corresponding to the REST/NRSF binding motif on the DNA (NRSE-ODN; *p *= 0.05). REST/NRSF binding in the KA-SE NRSE-ODN group did not significantly differ from binding in the controls *p *= 0.28 (Student's *t* test).

SE, induced by KA or experimental febrile seizures, increases nuclear accumulation of REST/NRSF and the binding of this transcription factor (TF) to the chromatin (McClelland et al., 2011a, 2014; Patterson et al., 2017). The administration of 10 nmol NRSE-ODN per hemisphere has been reported to block REST/NRSF chromatin binding (McClelland et al., 2011a, 2014). However, this dose seemed to induce malaise in adult male rats, limiting our ability to record them long term, as described by McClelland et al. (2011a). Therefore, we opted to employ an NRSE-ODN at a lower dose, 5 nmol per hemisphere. To test for the efficacy of this approach we performed ChIP and measured the amount of REST/NRSF bound to chromatin, specifically to the REST/NRSF binding site (NRSE) of an established REST/NRSF target gene, *Hcn1*. As shown in Figure 2, compared with hippocampi from control rats, REST/NRSF binding at the HCN1-NRSE gene was increased eightfold in KA-SE rats given SCR-ODN at 48 h after the SE (CTL vs KA-SE SCR-ODN, *p *= 0.017). In contrast, administration of NRSE-ODN to KA-SE rats significantly prevented the augmented REST/NRSF binding to the chromatin (*F*_(2,9)_ = 5.19; *p *= 0.04; one-way ANOVA; CTL vs KA-SE NRSE-ODN; *p *= 0.28). These results indicated that the lower dose of NRSE-ODN sufficed to diminish the binding of REST/NRSF to regulatory sites of an NRSF “target gene” DNA. Notably, this dose was not associated with apparent ill effects on the rats, enabling prolonged continuous video-EEG monitoring.

### Blocking REST/NRSF function transiently increases the latency to the emergence of spontaneous epileptic seizures and reduces seizure burden

**Figure 3.**
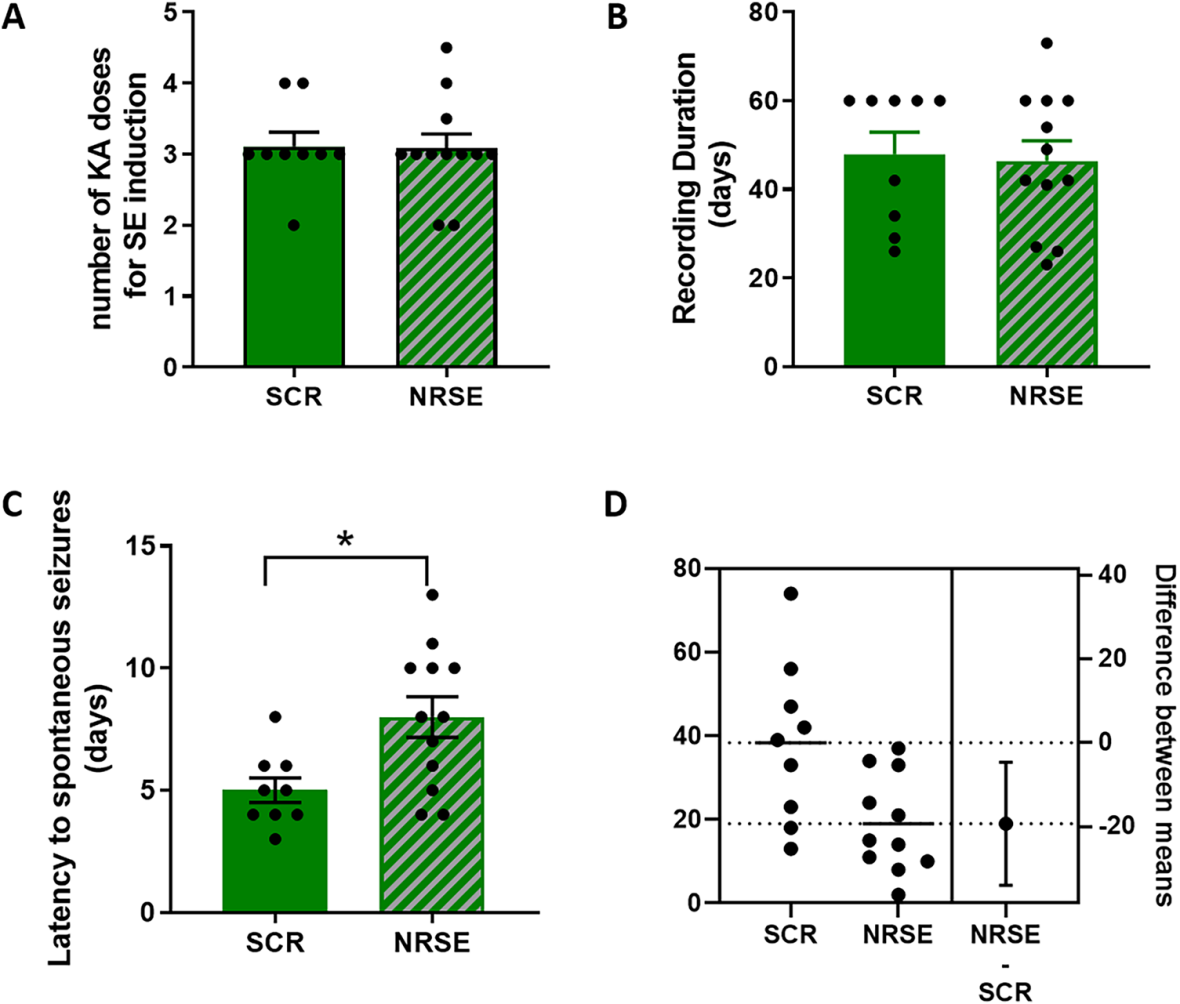
Transient interference with REST/NRSF function following KA-SE increases the latency to the emergence of spontaneous epileptic seizures and reduces seizure burden. ***A***, The number of KA injections and the total doses required to maintain SE for 3 h in the SCR versus the NRSE groups did not differ. ***B***, The overall duration of continuous video-EEG recording did not distinguish the groups (average 48 d). ***C***, The latency to the onset of the first spontaneous seizure in KA-SE rats receiving SCR-ODN averaged 5 d; in contrast, NRSE-ODN–receiving rats were seizure-free until the eighth day after the KA-SE (*p *= 0.0108). ***D***, An estimation plot for the data shown in ***C***. KA-SE NRSE-ODN *n* = 12; KA-SE SCR-ODN *n* = 9.

There were no differences in the number of KA doses (average 3, a total of 15 mg/kg) required to maintain SE for 3 h in the SCR versus the NRSE groups (Fig. 3*A*). We recorded KA-SE and control rats under continuous digital video-EEG for an average of 48 d (Fig. 3*B*) and compared rats given transient NRSE-ODN treatment (a dose on the SE day and a second 2 d later; *n* = 9) with the cohort given a SCR-sequence ODN (SCR-ODNs; *n* = 12). The latency to the onset of the first spontaneous seizure in KA-SE rats receiving SCR-ODN averaged 5 d; in contrast, NRSE-ODN–receiving rats were seizure-free until the eighth day after the KA-SE on average (*p *= 0.0108; Fig. 3*C*; estimation plot for this effect size is shown *n* 3D).

**Figure 4.**
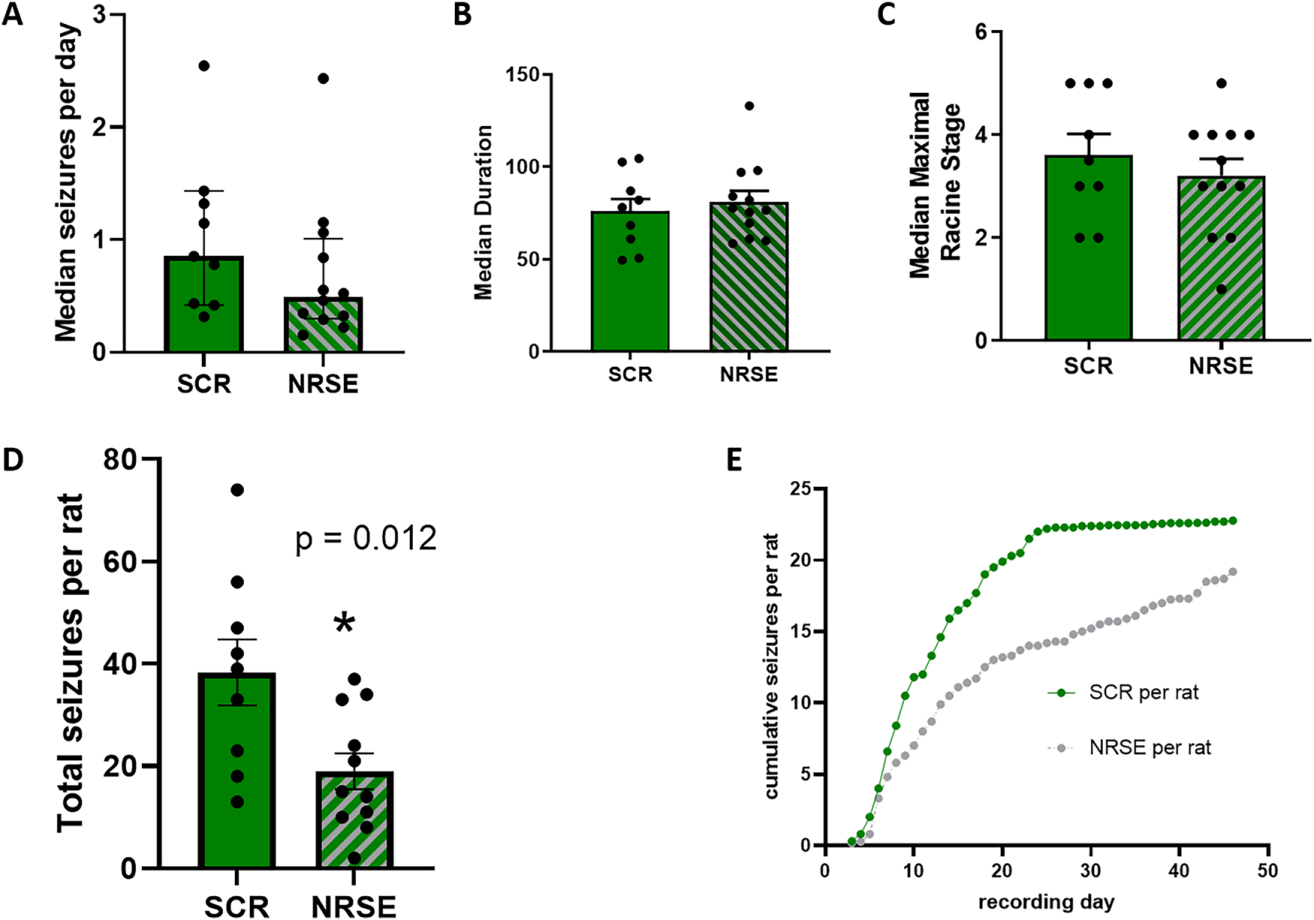
Transient interference with REST/NRSF function following KA-SE reduces the total number of spontaneous seizures and attenuates seizure cumulation with little effect on seizure severity or duration. ***A***, The median number of seizures per day was not significantly reduced in KA-SE rats receiving NRSE-ODNs (0.49; mean = 0.69 ± 0.18) compared with those receiving SCR-ODNs (0.85; mean = 1.028 ± 0.23; *p* = 0.16, Mann–Whitney test). Similarly, median seizure duration, and median seizure severity as determined by the Racine scale, did not distinguish the groups (***B*, *C***). ***D***, Blocking REST/NRSF function by two injections of a NRSE-ODN during the first 48 h following KA-SE reduced the total number of seizures of NRSE-ODN rats and attenuated the average cumulation of seizures per rat over the recording period. ***E***, A curve showing average cumulative seizures in the KA-SE SCR-ODN group (green) and the KA-SE NRSE-ODN group. A KS frequency analysis suggests that the group differ: KS distance, 0.25 (*p* < 0.0001) in SCR-ODN; KS distance, −0.166 (*p* = 0.0037) in NRSE-ODN rats. Area under the curve analysis: total area, 777 and 545 in the SCR-ODN and NRSE-ODN groups, respectively.

Aside from the latency to the onset of the first seizure, we quantified the average and median numbers of seizures per day, median seizure duration, and median seizure severity as determined by the Racine scale. Blocking REST/NRSF function transiently after KA-SE did not significantly influence these parameters, though there were weak trend for a reduction in both average seizure numbers (0.69/day in KA-SE NRSE and 1.028 in KA-SE SCR; *p* = 0.27) and median (0.86 and 0.49, respectively; *p* = 0.16; Mann–Whitney test; Fig. 4*A*–*C*). The intervention did reduce the cumulative average number of seizures per rat in NRSE-ODN rats over the same recording period as SCR-ODN rats: a Kolmogorov–Smirnov (KS) frequency analysis suggests that the group differs: KS distance, 0.25 (*p* < 0.0001) in SCR-ODN, and KS distance, −0.166 (*p* = 0.0037) in NRSE-ODN rats. In addition, the cumulative number (area under the curve) analysis showed the total area was 777 and 545 in the SCR-ODN and NRSE-ODN groups, respectively, that is, an attenuated cumulative seizure number of each rat.

Together, these experiments indicate that the augmented REST/NRSF chromatin binding induced by SE facilitates epileptogenesis. Accordingly, transient interference with this REST/NRSF function delays epilepsy development and modestly attenuates seizure burden, at a minimum over 7 weeks.

## Discussion

The principal findings of these experiments are as follows: (1) SE augments REST/NRSF chromatin binding, and it is possible to block this increase using NRSE-ODNs that are well tolerated; (2) transient reduction of REST/NRSF chromatin binding increases the latency to the first spontaneous seizure in the epileptogenic process triggered by SE; and (3) acute KA-SE–induced increase of REST/NRSF binding to the chromatin is not required for the consequent epilepsy, although it may accelerate epileptogenesis and increase the overall seizure burden.

We identify here the augmented REST/NRSF chromatin binding instigated by KA-SE in adult rats, first shown in 2011 and documented by several groups (Navarrette-Modesto et al., 2019; Chmielewska et al., 2020; Ghanem et al., 2021; Prestigio et al., 2021). REST/NRSF is also upregulated by SE provoked by experimental fever in developing rats (Brennan et al., 2016; Patterson et al., 2017). Importantly, in developmental SE, blocking REST/NRSF transiently sufficed to mitigate hippocampus-dependent memory deficits enduringly, suggesting the REST/NRSF chromatin binding plays a key role in SE-related maladaptive plasticity within hippocampal circuits. In support of this notion, augmented REST/NRSF chromatin binding was found in the hippocampus following significant early-life adversity/stress that results in serious adult memory problems (Bolton et al., 2020), and in that paradigm, a transient interference with REST/NRSF chromatin binding rescued normal memory enduringly (ibid). Thus, in the developing brain, REST/NRSF may play important roles in the epigenetic processes resulting from a variety of insults, potentially in accord with the unique roles of this factor during development (Ballas and Mandel, 2005; Gao et al., 2011; McClelland et al., 2011b; Nechiporuk et al., 2016).

In the current experiments, we demonstrate an increased latency and a modest attenuation of epileptogenesis after blocking REST/NRSF function subsequent to a defined proepileptogenic insult. These findings highlight the multifactorial nature and the complexity of epileptogenesis that follows insults (Committee, 2012; Löscher, 2012; EpiPM, 2015; Simonato et al., 2021). Numerous processes are clearly set in motion concurrently by an insult such as SE, neuronal death (although modest in the KA-SE paradigm used here; Dudek et al., 2002), and the SE itself triggers inflammatory mediators (Vezzani et al., 2011; Dedeurwaerdere et al., 2012; Aronica et al., 2017; Klein et al., 2018), and blood–brain barrier breaches further promote inflammatory processes (ibid). Metabolic changes may persist (Walker et al., 2016; Hall et al., 2017; Rho and Boison, 2022), and several epigenetic pathways within neurons lead to enduring changes of gene expression repertoires and the firing and connectivity of neuronal ensembles. REST/NRSF is very likely a member of this SE-induced “orchestra,” because blocking its function prolonged latency to epilepsy and reduced overall seizure burden, in accord with recent work using selective deletion of the TF in hippocampal excitatory cells (Natali et al., 2023). However, the current study indicates that the function of this TF in itself during the acute phase of epileptogenesis that follows SE is not fundamentally required for adult hippocampal epileptogenesis.

The current study examined whether a transient block of REST/NRSF binding to the chromatin aborts a proepileptogenic process and did not assess the potential role of chronic attenuation of REST/NRSF function in epileptogenesis. The upregulation of REST/NRSF expression that follows SE lasts at least a week (McClelland et al., 2014), while the precise half-life and duration of ODN effects is not fully resolved yet appears to be a week or shorter (Patterson et al., 2017). Thus, it is conceivable that chronic treatment with NRSE-ODNs will have more lasting or profound effects on epileptogenesis. A second limitation of the current study is the inclusion of male rats only: although epileptogenesis takes place in both sexes, it is theoretically possible that the role of REST/NRSF in this process is modulated by sex.

What might be the evolutionary purpose of augmented function of REST/NRSF, a transcriptional repressor, after seizures or other neuronal insults? Analyses of the specific genes targeted and suppressed by REST/NRSF in the hippocampus demonstrate a preponderance of genes that specifically define neuronal function, such as ion channels and glutamate and GABA receptor subunits. Neuronal functions, as compared with the functions of many other cell types, are highly energy intensive: the maintenance of the resting membrane potential and the firing of neurons require major energy expenditures (up to 50% of the total body energy utilization). Therefore, in the face of major insults associated with “energy crises” such as SE (massive demand), stroke (plummeting supply), or chronic stress (increased demand and reduced supply), it is logical for a neuron to repress the expression of neuronal genes and aim to survive as a more quiescent cell. Augmented REST/NRSF function accomplishes this strategy.

In conclusion, the transcriptional repressor REST/NRSF contributes crucially to hippocampal plasticity that follows insults in the developing hippocampus. In the adult, interference with the augmented REST/NRSF function that follows proepileptogenic insults delays epileptogenesis and moderately reduces seizure burden.

## Synthesis

Reviewing Editor: Viji Santhakumar, University of California Riverside Decisions are customarily a result of the Reviewing Editor and the peer reviewers coming together and discussing their recommendations until a consensus is reached. When revisions are invited, a fact-based synthesis statement explaining their decision and outlining what is needed to prepare a revision will be listed below. The following reviewer(s) agreed to reveal their identity: Nicholas Poolos.

The manuscript examines the antiepileptogenic potential of transient inhibition of the REST/NRSF transcription factor, timed shortly after status epilepticus (SE) using a kainic acid seizure induction model in rats. The primary effects are increased latency to spontaneous seizures and an overall reduction in total seizure burden, although the duration and severity of spontaneous seizures were not affected. The results are interesting and suggest that targeting REST/NRSF immediately after SE may provide at least some antiepileptogenic potential, although epileptogenesis was not fully prevented in any of the animals. The authors have made several revisions including clarification of methods which strengthen the manuscript. Few additional clarifications are needed.

1. The rationale provided for justifying the use of only male rats is not convincing. The provided justification is that a prior study only used male rats, so this subsequent study should also use male rats. Taken to the logical extension, this rationale would justify only ever studying male animals since the vast majority of prior work in neuroscience only used males. At the very minimum, a discussion of the sole use of males as a limitation of the work should be included in the text.

2. Although the new addition of EEG traces is appreciated, the presentation requires improvement for inclusion in a publication. The two example traces appear to only be screenshots from different recording/analysis programs. This presentation does not meet the standard for publication and needs to be appropriately revised.
